# Identification and Expression Analysis of R2R3-MYB Family Genes Associated with Salt Tolerance in *Cyclocarya paliurus*

**DOI:** 10.3390/ijms23073429

**Published:** 2022-03-22

**Authors:** Zijie Zhang, Lei Zhang, Yang Liu, Xulan Shang, Shengzuo Fang

**Affiliations:** 1 College of Forestry, Nanjing Forestry University, Nanjing 210037, China; iszhangzj@sina.com (Z.Z.); zhanglei321@njfu.edu.cn (L.Z.); lyang_188@sina.com (Y.L.); shangxulan@njfu.edu.cn (X.S.); 2 Co-Innovation Centre for Sustainable Forestry in Southern China, Nanjing Forestry University, Nanjing 210037, China

**Keywords:** wheel wingnut, R2R3-MYB transcription factors, salt stress, qRT-PCR, phytohormone signal

## Abstract

R2R3-MYB transcription factors are most abundant in the MYB superfamily, while the *R2R3-MYB* genes play an important role in plant growth and development, especially in response to environmental stress. *Cyclocarya paliurus* is a multifunction tree species, and the existing resources cannot meet the requirement for its leaf production and medical use. Therefore, lands with some environmental stresses would be potential sites for developing *C. paliurus* plantations. However, the function of *R2R3-MYB* genes in *C.* *paliurus* in response to environmental stress remains unknown. In this study, to identify the roles of *R2R3-MYB* genes associated with salt stress response, 153 *CpaMYB* genes and their corresponding protein sequences were identified from the full-length transcriptome. Based on the comparison with MYB protein sequences of *Arabidopsis thaliana*, 69 R2R3-MYB proteins in *C. paliurus* were extracted for further screening combined with conserved functional domains. Furthermore, the MYB family members were analyzed from the aspects of protein sequences alignment, evolution, motif prediction, promoter cis-acting element analysis, and gene differential expression under different salt treatments using both a pot experiment and hydroponic experiment. The results showed that the *R2R3-MYB* genes of *C.* *paliurus* conserved functional domains, whereas four *R2R3-MYB* genes that might respond to salt stress via regulating plant hormone signals were identified in this study. This work provides a basis for further functional characterization of R2R3-MYB TFs in *C. paliurus*.

## 1. Introduction

Plants, as sessile organisms, often experience various abiotic stresses such as salt, waterlogging, drought, and cold and can respond to various environmental stresses through a series of defense mechanisms [1,2]. Among the environmental stress, soil salinization is one of the main factors that restricts plant production [3,4]. Salt stress induces the disintegration of cell membrane, the production of reactive oxygen species, the weakening of metabolic processes, and the inhibition of photosynthesis and nutrient absorption, thus delaying the growth and development of plants and reducing the quality of agricultural and forestry crops [4]. Under present and expected climate change scenarios, soil autonomous salinization via seepage of saline groundwater rather than enhanced seawater intrusion is a threat to the agricultural sustainability and more salinization areas would appear [5,6]. Hence, it is necessary to study the regulation mechanism of plant salt tolerance for selecting salt-tolerant species or genotypes.

Transcription factors (TFs), which regulate gene expression as the molecular switch, are involved in plant responses to abiotic stresses by combining cis-acting elements of stress-related genes [7]. In general, TFs consist of four domains, including a transactivation domain, a DNA binding domain, a nuclear localization signal, and oligomeric sites [8]. Different DNA binding domains determine the specificity of TFs, which can be divided into various families because of different types of conserved domains, such as WRKY, AP2/ERF, MYB, and bZIP [9]. As one of the largest transcription factors in plants, MYB TFs possess a conserved DNA-binding domain constituted by one to four conserved repeats, whereas MYB superfamily genes can be divided into four types by reason of different incomplete MYB tandem repeat numbers [8,10]. MYB family genes have been identified in a large number of plants and the number varies among plants, such as *Liriodendron chinense* (204 *MYB* genes) [11], *Avicennia marina* (185 *MYB* genes) [12], *Casuarina equisetifolia* (182 *MYB* genes), and peach (*Prunus persica*) (155 *MYB* genes) [13,14]. Meanwhile, specific functions of MYB TFs including regulating development [15,16,17] and secondary metabolism [18], hormone signal transduction, and responding to abiotic stress have also been studied [19,20].

R2R3-MYBs as the largest family of MYB TFs in plants have various biological functions, especially in response to environment stress. Zhao et al. reported that transgenic tobacco with overexpressed *PsnMYB108* was shown to have greater ability of salt tolerance than wild-type tobacco [21]. Moreover, a novel gene of *TaMYB sdu1*, which could improve tolerance to drought and salt stress, was detected in wheat (*Triticum aestivum*) [22]. Many studies have indicated that *AtMYB2*, *AtMYB44*, and *AtMYB74* in *Arabidopsis thaliana* play a vital role in enhancing the tolerance to salt stress [23,24,25], while *TaMYB34* in transgenic tobacco was involved in drought, high temperature, and high salt stress [26]. Analysis of MYB family factors is helpful to explore the response mechanism of plants under abiotic stress. However, the MYB family has not been systematically identified and studied in most plants due to the lack of high-quality reference genomes. Fortunately, the third generation of transcriptome sequencing (Pacbio single-molecule sequencing) technology provides a reference for many species lacking genomes, which can solve the problems in the analysis of these species families to a certain extent [27].

Wheel wingnut (*Cyclocarya paliurus*) is a multifunction tree species with high values of medicinal function, timber production, and landscape and is mainly distributed in subtropical mountain districts of China [28,29]. Especially, its leaves enrich bioactive substances that are effective in preventing hyperglycemia and diabetes [30,31]. However, most existing resources of *C. paliurus* are found in natural forests and their quantity cannot meet the requirement for its leaf production and medical use, which seriously affects the development and utilization process of *C. paliurus*. Therefore, developing *C. paliurus* plantation with oriented cultivation is the best option for leaf production [3,31]. Owing to the limitation of land resources in China, coastal saline would be a potential land resource for developing *C. paliurus* plantations. Some studies have explored the strategies of external ions alleviating sodium toxicity and the transcriptional regulatory network in *C. paliurus* seedlings under salt stress [3,32], whereas the response of MYB gene family in the species to salinity still remains unknown. The objective in this study is to assess whether *R2R3-MYB* genes are involved in the response to salt stress and identify R2R3-MYB family genes associated with salt tolerance in *C. paliurus* based on the analysis of full-length transcriptome sequences and their expression profile. Results from this study would provide an essential basis for improving the salt tolerance in *C. paliurus* at the molecular level and the possibility to develop *C. paliurus* resource in the coastal saline areas.

## 2. Results

### 2.1. Identification of R2R3-MYB Genes and Prediction of Encoded Proteins

Based on the full-length transcriptome sequences of *C. paliurus*, 211 *CpaMYB* genes and their corresponding protein sequences were identified. Subsequently, according to the Hidden Markov model (Pfam: PF00249) of MYB domain, 211 sequences of *C. paliurus* and *A. thaliana* protein sequences were homologous and retrieved. After removing redundant sequences, 153 MYB protein sequences including 69 R2R3-MYB sequences of *C. paliurus* with typical complete MYB domain were obtained ultimately. Meanwhile, the corresponding DNA sequences and CDS sequences of 69 *MYB* genes were also extracted, laying a foundation for further study. As shown in Table S1, the physical and chemical properties of the 69 R2R3-MYB proteins were forecasted. The relative molecular weight (*Nw*) ranged from 12606.55 Da (Cpa033006) to 60299.14 Da (Cpa093839), whereas the isoelectric point (*pI*) ranged from 4.86 (Cpa050782) to 10.53 (Cpa002553). The isoelectric points of 39 *R2R3-MYB* proteins were less than 7.0, implying that half of the *R2R3-MYB* proteins were acidic. Furthermore, the results of protein subcellular revealed that all 69 members were located in the nucleus (Table S1).

### 2.2. Phylogenetic Analysis of R2R3-MYB Family Genes

**Figure 1:**
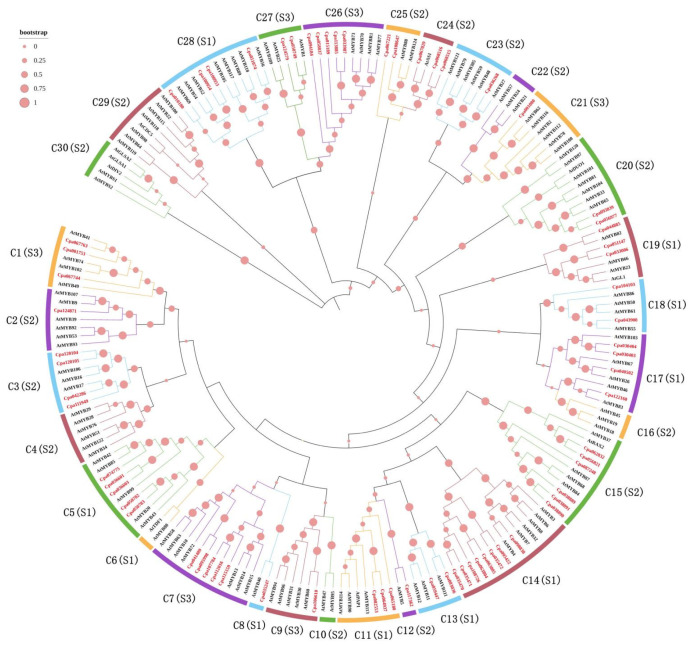
Phylogenetic tree and classification of R2R3-MYB subfamily proteins in *A. thaliana* and *C. paliurus*. The number of MYB proteins of *A. thaliana* and *C. paliurus* is 120 and 69, respectively. The red dots represent boot values—the larger the dot, the larger the bootstrap value. The names of genes typed in red represent CpaMYBs and those in black represent AtMYBs. S1–S3 represent three subgroups and C1–C30 represent 30 clades of the tree. For example, C5 (S1) indicates that these MYBs are grouped into C5 clade, which belongs to S1 subgroup.

In order to explore the phylogenetic relationships, the phylogenetic tree of *C. paliurus* was constructed using 69 CpaMYB proteins and 120 AtMYB proteins based on the alignment (Figure 1). In accordance with the classification of *R2R3-MYB* genes in *A. thaliana* [8], the phylogenetic tree could be classified into three subgroups, named S1–S3. Possible functions of S1, S2, and S3 subgroups were the regulation of secondary metabolism, the regulation of cell morphogenesis and organ development, and the mediation of signal transduction pathways in response to abiotic stress and pathogen attack, respectively. According to results from Dubos et al. [8], we inferred that CpaMYB proteins in the same clade would have similar functions, and CpaMYBs were further divided into 30 clades (designated C1-C30) according to the similarity of protein sequences and the high bootstraps of clustering results. Possible functions of 30 clades are given in Table S2. In this study, 17 *R2R3-MYB* genes involved in stress response were identified in the third subgroup (S3), including C1, C7, C9, C21, C26, and C27. Based on the results of gene functional prediction, *CpaMYB* genes may exhibit a wide range of functions and play an important role in response to biological stress.

### 2.3. Gene Structure and Conserved Motifs Analysis of R2R3-MYB Proteins

**Figure 2:**
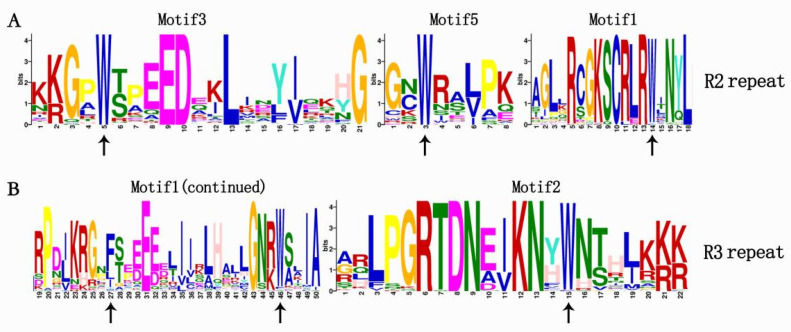
R2 and R3 MYB repeats across 69 R2R3-MYB proteins in *C. paliurus*. The sequence logos of the R2 (**A**) and R3 (**B**) MYB repeats are based on alignments of 69 *C. paliurus* R2R3-MYB domains. The bit score represents the information for each position in the sequences. Arrows represents the conserved tryptophan residues in the MYB domain.

**Figure 3:**
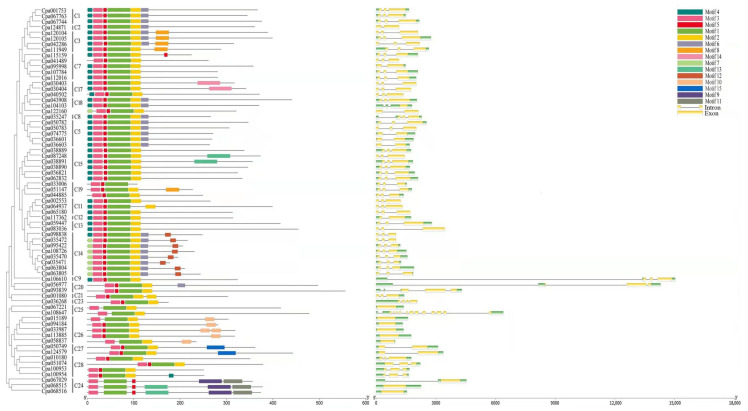
Phylogenetic relationship (**left**), motif distributions (**middle**), and exon/intron gene structures (**right**) of *C. paliurus R2R3-MYB* genes. Fifteen motifs were represented by fifteen kinds of colored blocks. The length of the gray line represents the length of a sequence relative to that of all the other sequences. The position of each block represents the location of the motif.

To examine the features of homologous domains and the level of conservation of R2 and R3 repeats in the R2R3-MYB proteins of *C. paliurus*, the online MEME (Multiple Em for Motif Elicitation) tool was used to search for conserved motifs and to download the sequence logos (Figure 2). In order to clearly see the order and classification of the MYB proteins, the motif data are arranged according to phylogenetic tree results (Figure 3). We identified 15 distinct motifs in the 69 R2R3-MYB proteins, where 4 were highly conserved motifs and presented in 61 of the 69 R2R3-MYB proteins (Figure 3). The four conserved motifs were motif 3, motif 5, motif 1, and motif 2, which contained 21, 8, 50, and 11 amino acids, respectively. The sequence logos showed that motifs 3 and 5 as well as the front part of motif 1 were composed of the R2 repeat, whereas the back part of motif 1 and all of motif 2 were composed of the R3 repeat (Figure 2). As shown in the sequence logos (Figure 2), three separate triplet tryptophan residues (W) were present in R2 repeat, which constitute helix-turn-helix (HTH) structure with glycine (G) and leucine (L) at the seventh and eighth positions after the second W and conduce to sequence-specific binding of DNA. Further, R2 and R3 repeats contained highly conserved groups of glutamic acid (E)-glutamic acid (E)-aspartic acid (D) residues (EED) and glutamic acid (E)-glutamic acid (E)-glutamic acid (E) residues, which is consistent with previous studies in poplar [33], sweet orange [34], and peach [14]. Figure 3 also showed that Cpa067029, Cpa068515, and Cpa068516 clustered in the C24 clade had unique motifs, including 1, 3, 5, 9, 11, and 13 in R2R3-MYB proteins of *C. paliurus*, which is similar to the result from Chinese jujube [10].

In order to gain more in-depth knowledge of the structural diversity of MYB TFs, the exon/intron organization of *R2R3-MYB* genes in *C. paliurus* was used to investigate further, and a gene structure diagram of these 69 *R2R3-MYB* genes was constructed (Figure 3). It was found that the clustering pattern of the *CpaMYBs* was consistent with the exon/intron structures in the 13 subgroups, such as subgroups C3, C14, C15, C17, and C26 (Figure 3). However, there are also exceptions in the four subgroups, which contain different members of introns, such as subgroup C5, C7, C18, and C25. Exon/intron analysis showed that the coding regions of the 69 *R2R3-MYB* genes were interrupted by introns, whereas the 69 genes were highly diverse in terms of their structure, including the number and relative positions of introns and exons. A large number of *CpaR2R3-MYB* genes contained introns, except two genes (*Cpa068515* and *Cpa068516*) from the subgroup C24, one gene (*Cpa067221*) from the subgroup C25, and five genes (*Cpa015189*, *Cpa094184*, *Cpa033987*, *Cpa113885*, and *Cpa058837*) from the subgroup C26. Figure 3 also showed that the number of introns ranged from 0 to 3 according to the *R2R3-MYB* gene structure, except for two genes (*Cpa093839* and *Cpa108647*), which had 4 and 12, respectively. More than half of the *CpaMYB* genes had two exons. To sum up, the dependability of our grouping classifications was strongly supported by similar gene structures and phylogenetic tree groupings.

### 2.4. Promoter Motif Analysis

**Figure 4:**
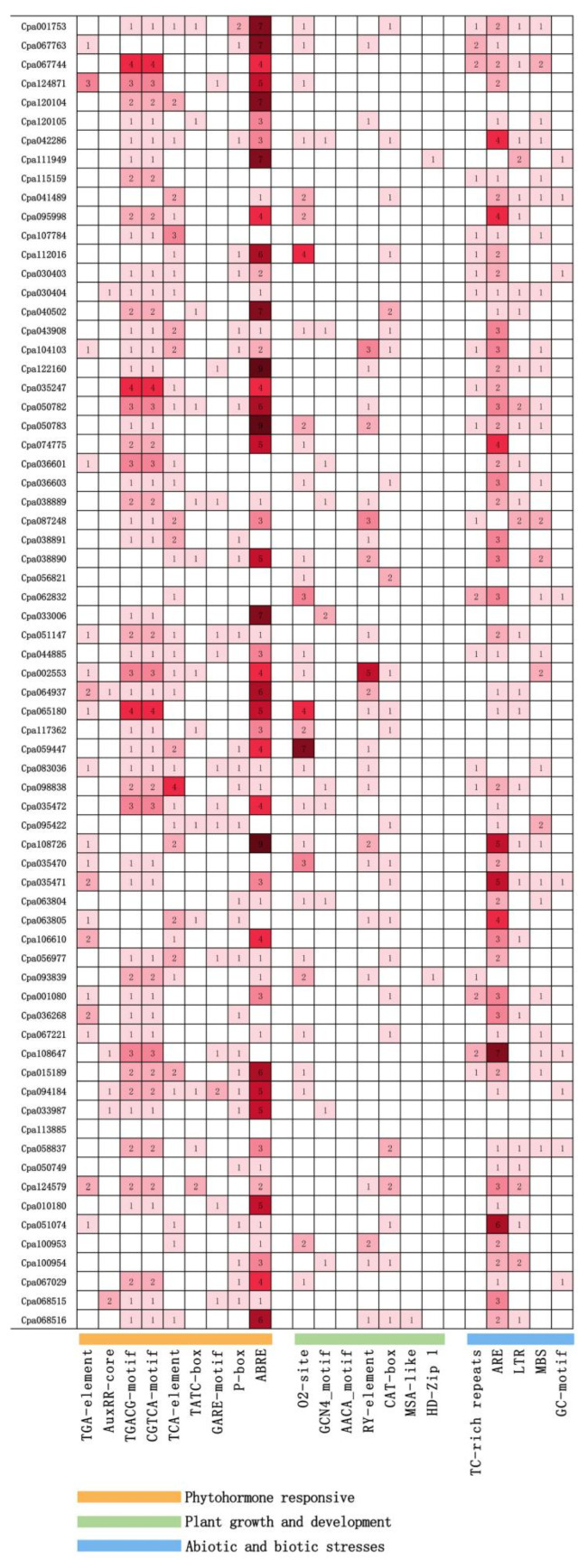
Cis-acting elements analysis of 69 *CpaMYB* genes in promoter region. Numbers in the squares showed the number of each cis-acting element in the promoter region. The darker the squares, the greater the number.

The conserved motifs contained in promoter regions of genes are important to the regulation of gene expression and biological processes in plants. Thus, conserved motifs in the promoter region were identified in 69 *R2R3-MYB* genes of *C. paliurus* to further understand their function in abiotic stress tolerance (Figure 4).

A large number of cis-regulatory elements (CREs) were detected and can be divided into three subgroups associated with phytohormone response, plant growth and development, and stress responses, respectively (Figure 4). Among the first subgroup of cis-regulatory elements (CREs), the ABA-responsive element (ABRE) was the most abundant, which was detected in the promoters of 56 *CpaMYB* genes with the ABRE number of 214 in total. Following was the MeJA-responsive element (CGTCA motif and TGACG motif), which was observed in 54 *CpaMYB* genes with the total number of 88. However, only six *CpaMYB* genes contained the AuxRR-core, which is an auxin-responsive element.

Cis-elements-related growth and development included the zein metabolism regulation element (O2-site), meristem expression element (CAT-box), cell cycle regulation element (MSA-like), seed-specific regulation element (RY-element), and endosperm expression element (GCN4_motif). Additionally, the cell cycle regulation (MSA-like) element was also found in *Cpa068516* promoter, while the differentiation element of the palisade mesophyll cells (HD-Zip1) was observed in both *Cpa093839* and *Cpa111949* promoters.

In the present study, five stress-related cis-elements were also identified (Figure 4), including the anaerobic-induced response element (ARE), the drought-induced response element (MBS), the low-temperature response element (LTR), the anaerobic-induced response element (ARE), and the defense and stress-response element (TC-rich) were all found in the promoters of 57, 28, 28, and 20 *CpaMYB* genes. These results suggest that *CpaMYB* genes would regulate abiotic stress and phytohormone signaling and play a vital role in plant growth and development.

### 2.5. Profiling of Expressed CpaMYB Genes and Verification of Selected Gene Expression

**Figure 5:**
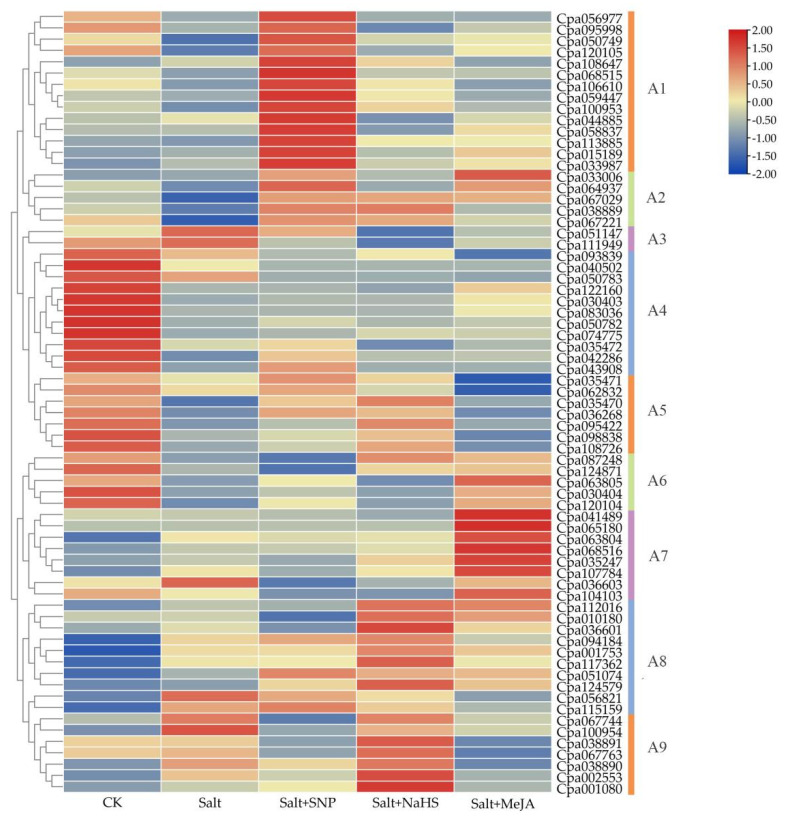
Clustering expression analysis of 69 *CpaMYB* genes in different salt stress treatments based on the pot experiments. The CK (control), Salt (0.4% NaCl), Salt+SNP (0.4% NaCl with 0.25 mM SNP), Salt + NaHS (0.4% NaCl with 0.5 mM NaHS), and Salt + MeJA (0.4% NaCl with 0.2 mM MeJA) represent the five different treatments. The transcript abundance level was normalized and hierarchically clustered by using the log 2 (FPKM + 1) comparison among genes of different treatments. The expression value is presented on the color scale, with red representing high expression and blue representing low expression. A1–A9 represent different clusters. In order to distinguish A1–A8 clusters more intuitively, lines of different colors were used on the right.

Gene expression profiling can provide important clues for determining gene function. Based on transcriptome data from the pot experiment, heat maps of 69 genes in different treatments consisting of CK (control), Salt (0.4% NaCl), Salt + SNP (0.4% NaCl with 0.25 mM SNP), Salt + NaHS (0.4% NaCl with 0.5 mM NaHS), and Salt + MeJA (0.4% NaCl with 0.2 mM MeJA) were analyzed after 30 days of the treatments. The results showed that the expression of 75.36% (57/69) *MYB* genes was induced/repressed under two concentrations of CK and NaCl treatments (Figure 5). Salt stress had a noticeable effect on the expression of *CpaR2R3-MYB* genes. Among the 69 *CpaMYB* genes, *Cpa001753* (more than 23-fold), *Cpa115159* (more than 12-fold), *Cpa094184* (more than 11-fold), *Cpa107784* (more than 9-fold), *Cpa117362* (more than 4-fold), *Cpa112016* (more than 2.2-fold), *Cpa067744* (more than 2-fold), *Cpa063804* (more than 1.9-fold), and *Cpa033987* (more than 1.3-fold) were strongly upregulated in the NaCl treatment compared with CK. However, most of the genes showed different expression patterns in the other three treatments (Salt + SNP, Salt + NaHS, and Salt + MeJA) due to the addition of different exogenous mitigating substances (Figure 5).

According to their diverse expression patterns, the 69 *CpaMYB* genes were gathered into nine clusters, named A1–A9 (Figure 5). A number of *CpaMYB* genes in cluster A1 strongly and preferentially expressed under the Salt+SNP treatment but expressed moderately in the other four treatments (Figure 5). However, *CpaMYBs* in cluster A2 were only strongly expressed under the three salt treatments with exogenous substance addition (Salt + SNP, Salt + NaHS, and Salt + MeJA). The highest transcript levels for *CpaMYB* genes in cluster A4 were detected in CK treatment, while very low expression levels of these genes were observed in the other four treatments. In contrast, *CpaMYB* genes in cluster A8 were induced and kept at a high level under the salt stress treatments, while very low expression of these genes was observed in the CK. The genes in cluster A5 showed a very low expression level in salt + MeJA treatment, whereas the genes in clusters A6 and A7 had much higher expression in this treatment. *CpaMYBs* in cluster A9 were mainly expressed in salinity condition and Salt + NaHS treatment (Figure 5).

**Figure 6:**
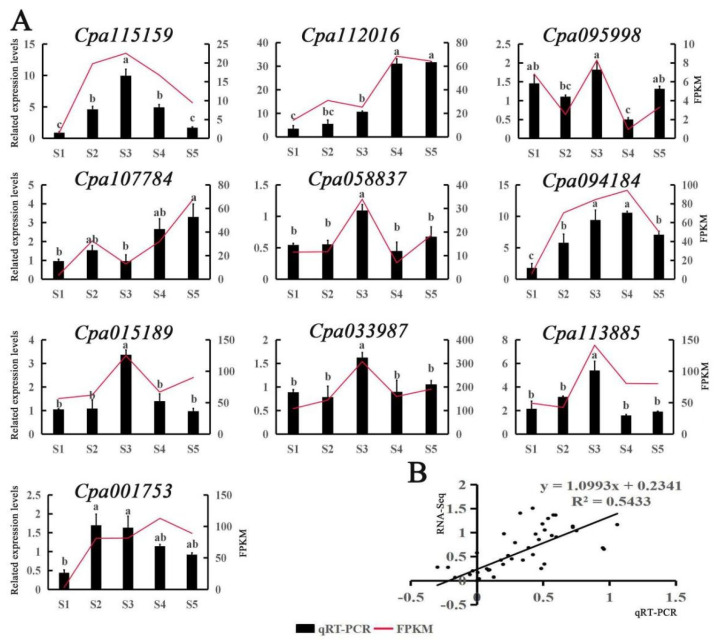
qRT-PCR validation of the transcriptome data results for 10 selected genes. S1–S5 represent CK, Salt, Salt + SNP, Salt + NaHS, and Salt + MeJA treatments, respectively. (**A**) Expression levels of 10 genes and FPKM values. The red folds represent FPKM values in transcriptome data, and the vertical bars shown in the columns represent relative expression level by qRT-PCR. Different letters denote significant differences according to Tukey’s test (*p* < 0.05). (**B**) Correlation analysis of the gene expression ratios between qRT-PCR and FPKM.

To verify the results of transcriptome sequencing from the pot experiment, we randomly selected 10 genes from transcriptome data for qRT-PCR validation (Figure 6). Notably, each gene showed differential responses to the various treatments (Figure 6A). For example, compared with CK, three genes (*Cpa115159/094184/001753*) were significantly upregulated in the salt treatment (0.4% NaCl), while 5 genes (*Cpa115159/058837/015189/033987/113885*) showed an expression spike in the Salt+SNP treatment (Figure 6A). Furthermore, no significant difference was detected in the expression of seven genes (*Cpa112016/107784/058837/015189/033987/113885/001753*) between Salt + NaHS and Salt + MeJA treatments. The results showed that the expression trend of qRT-PCR values was basically consistent with that of FPKM values (Figure 6A). Furthermore, correlation analysis indicated that the correlation coefficient (R^2^) reached 0.5433 (Figure 6B), suggesting that the transcriptome data were accurate and reliable, and could be used in subsequent experiments.

### 2.6. Profiling of Expressed CpaMYB Genes in Hydroponic Experiment

**Figure 7:**
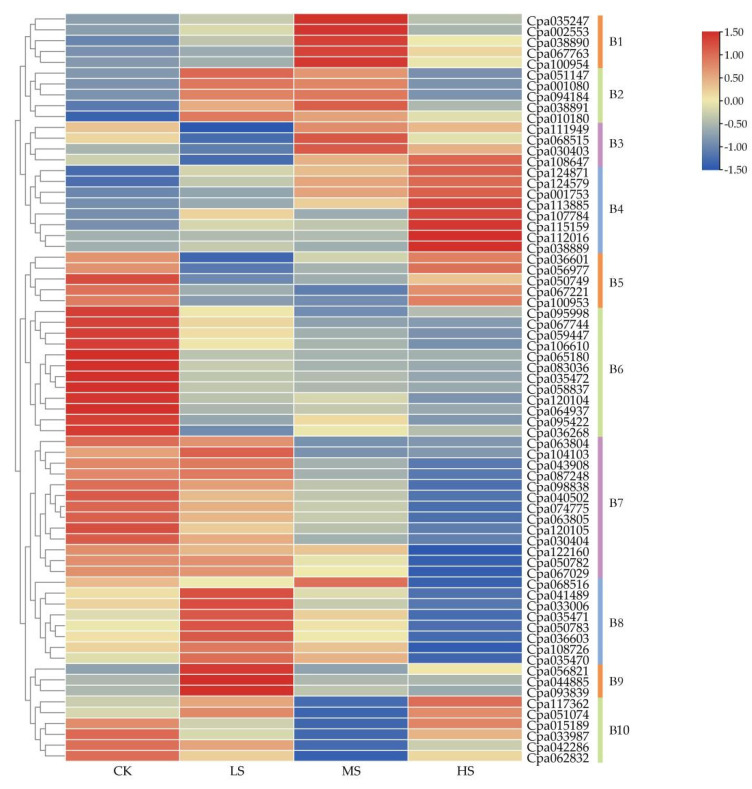
Clustering expression analysis of 69 *CpaMYB* genes in salt stress based on hydroponic experiments. The CK, LS, MS, and HS represent NaCl concentrations of 0%, 0.15%, 0.3%, and 0.45%, respectively. The transcript abundance level was normalized and hierarchically clustered by using the log 2 (FPKM + 1) comparison among genes of different treatments. The expression value is presented on the color scale, with red representing high expression and blue representing low expression. B1–B10 represent different clusters. In order to distinguish B1–B10 clusters more intuitively, lines of different colors were used on the right.

In order to further verify the expression pattern of *CpaR2R3-MYB* genes under salt stress, a hydroponic experiment with four salt concentrations (0%, 0.15%, 0.3%, and 0.45% NaCl) was conducted to compare the results from the pot experiment. Based on the transcriptome data, heat maps of 69 genes in different treatments were analyzed after 30 days of the treatments (Figure 7). The result showed that the expression of 69 *MYB* genes was induced/repressed under different concentrations of NaCl treatments, confirming that the expression of *MYB* genes was significantly affected by salt stress.

According to their diverse expression patterns, the 69 *CpaMYB* genes were clustered into 10 groups (Figure 7). The genes in cluster B2 and B3 were mainly expressed in the middle (0.30% NaCl) salinity condition and did not change significantly under other treatments, whereas genes in cluster B10 had significantly lower expression in the middle salinity condition. *CpaMYBs* in cluster B2 were slightly upregulated in low and middle salinity conditions. Most genes in cluster B6 had a very low level of expression under all salt treatment, while the genes in cluster B7 were significantly downregulated as salinity intensity increased. However, expressions of most genes in cluster B4 varied with salt concentration treatments, and expression of these genes were all upregulated under salt treatments and peaked at a high salinity treatment (0.45% NaCl) (Figure 7). Compared with the CK, the expressions of *Cpa001753*, *Cpa115159*, *Cpa107784*, *Cpa113885*, and *Cpa112016* genes in cluster B4 were upregulated by nearly folds of 65, 35, 26, 22, and 3.4 in the high salinity treatment (0.45% NaCl), respectively, suggesting that these genes may play an important role in improving the salt tolerance of *C. paliurus*.

## 3. Discussion

### 3.1. Characterization of the R2R3-MYB Gene Family in C. paliurus

The MYB transcription factor family is one of the largest families in plants, while *R2R3-MYB* genes are involved in environmental response and stress response [8]. However, there are no systematic researches of MYB gene family in *C. paliurus*; therefore, this is the first time that the *R2R3-MYB* gene family has been identified and analyzed using bioinformatics tools and the full-length transcriptome sequences of *C. paliurus*. In this study, 69 *R2R3-MYB* genes were identified (accounting for 45.10% of all 153 MYBs), which is similar to the MYB gene family of *Solanum tuberosum* [35] and *L. chinense* [36]. Indeed, *R2R3-MYBs* were a vitally important subgroup in MYB gene family and owned the largest number in many plants, such as *A. thaliana* [8], *Capsicum annuum* [37], and *O. sativa* [38]. The number of R2R3-MYB members identified in *C. paliurus* from this study was significantly less than 126 in *A. thaliana* and 109 in *O. sativa*, which may be due to the limitations of transcriptome data used in the study. We suggest that further research is needed after the genome of *C. paliurus* is published in the future.

Based on the phylogenetic tree of 69 CpaMYB proteins and 120 AtMYB proteins, our results showed that most clades contained in *C. paliurus* was similar to *A. thaliana* members (Figure 1), suggesting that the evolutionary process of *C. paliurus* is highly conservative. Meanwhile, all clades of *CpaMYB* genes contained *AtMYB* genes, indicating that the MYB transcription factor family was formed before the differentiation of *C. paliurus* in the evolutionary process. Moreover, some previous studies reported that *AtMYB77* might regulate the plant in response to drought and salt stress [39], whereas *AtMYB73* is a negative regulator of tolerance to salt stress [40]. Thus, we think five genes (*Cpa033987/113885/015189/094184/058837)* clustered in the same clade with *AtMYB77* and *AtMYB73* may respond well to salt stress in *C. paliurus*.

Diversity of gene structure and motifs is a vital part of gene family evolution, whereas the loss and gain of introns are the main reasons of gene structural diversity [41]. Therefore, the different gene structures of 69 *CpaMYB* genes are of great significance to the evolution of *C. paliurus*, which could help obtain new functions in the evolution of genes and better adapt to environmental changes [42]. Similar to the other studies [8,37,38], the motif distribution in the same subfamily showed a high degree of similarity in *C. paliurus* and most motifs were located at the N-terminal regularly, suggesting that these motifs have significant functions in MYB genes. However, a little number of motifs (such as motif 10 and motif 12) were scattered at the C-terminal, and these motifs may also have some specific functions. For example, motif 10 was only detected in five genes (*Cpa033987/113885/015189/094184/058837)* (Figure 3), inferring that motif 10 may perform functions in responding abiotic stresses.

### 3.2. Responses of R2R3-MYB Genes to Salt Stress in C. paliurus

The study of gene expression patterns can provide important clues for understanding gene function. It has been noted that some *R2R3-MY*B genes have vital regulatory functions in plant resistance to various abiotic stresses [43]. For example, *AmMYB1* expression confers better salt tolerance in transgenic tobacco plants [44] and *GHMYB108-like* may have important an regulatory function in response to drought and salt stress [45]. Recent studies have shown that overexpression of *VcMYB4a* enhanced the sensitivity of blueberry callus to salt stress [46]. Results from our pot experiment indicated that 75.36% (57/69) of *MYB* genes were induced/repressed under NaCl stress, whereas the expressions of nine genes were strongly upregulated (Figure 5). Interestingly, the abovementioned nine genes highly expressing under salt stress had similar expression levels in the NaCl treatments with three exogenous substance additions, indicating that these genes may act at all different salt concentrations (Figure 5). Furthermore, these genes all belonged to the third subgroup (S3) of the phylogenetic tree (Figure 1), suggesting that these genes may play important roles in response to salt stress.

To further confirm the expression patterns of *CpaR2R3-MYB* genes, a transcriptome sequencing analysis was also conducted under various NaCl concentrations after 30 days of treatments in the hydroponics. The gene expression analysis revealed that most genes in cluster B4 showed different expressions under various NaCl concentrations, where the expressions of these genes were dramatically affected by the high salt concentration (Figure 7). Interestingly, six (*Cpa001753*/115159/*107784*/*112016*/*113885*/*124579*) of all eight genes in this group were clustered in the S3 subgroup of the phylogenetic tree (Figure 1), suggesting that these six *CpaMYB* genes highly responded to high salt stress, in agreement with the results from tartary buckwheat [47]. However, after comprehensively analyzing the gene expression results from both the pot and hydroponic experiments, it was found that *Cpa001753*, *Cpa115159*, *Cpa107784*, and *Cpa112016* all showed highly specific expressions under salt stress. In addition, some previous studies reported that the expression of *AtMYB41* was associated with osmosis and salt stress [48] while *AtMYB74* had a positive regulation of transcriptional response under salt stress [25]. In the present study, *Cpa001753*, *Cpa067763*, *Cpa067744*, *AtMYB41*, and *AtMYB74* were clustered in the C1 clade in the phylogenetic tree (Figure 1), further confirming that *Cpa001753* in *C. paliurus* may play a key role in the response to salt stress.

### 3.3. Phytohormone Signal Response to Salt Stress

In plants, hormone signaling response to abiotic stress involves the interaction of several plant hormones such as ABA, SA, and MeJA, while R2R3-MYB TFs may be important participants [49,50]. Some studies have shown that ABA is usually the main signal to increase the transcription level of stress-responsive genes and induces the expression of genes that enhance the plant resistance to the abiotic stress [51,52]. It is known that *AtMYB13* and *AtMYB14* respond well to the treatments of ABA, SA, MeJA, and other hormones, which are closely related to plant development and response to biological stress [53,54,55]. For example, transgenic expression of *MYB15* gene in *A. thaliana* can improve its tolerance to drought and salt stress by increasing sensitivity to exogenous ABA [56]. However, the abiotic stress response signaling transduction pathways can be divided into two major forms, ABA-dependent and ABA-independent pathways [57]. Based on the classification of *R2R3-MYB* genes in *A. thaliana*, our results showed that 17 *CpaMYBs* in the S3 subgroup may be involved in stress response, whereas 10 of 17 *CpaMYBs* showed highly specific expressions under the salt stress (Figure 5 and Figure 7), inferring that these 10 genes (*Cpa001753/067744/107784/112016/115159/001080/033987/113885/094184/124579*) may carry out functions in response to salt stress. However, only four of ten genes showed strongly specific expressions in both hydroponic and pot experiments, suggesting that these four genes (*Cpa001753/107784/112016/115159*) may have a better response to salt stress.

The phylogenetic tree showed that *Cpa115159*, *Cpa107784*, *Cpa112016*, *AtMYB13*, *AtMYB14*, and *AtMYB15* were clustered in the C7 clade (Figure 1), indicating that the functions of *Cpa115159*, *Cpa107784*, and *Cpa112016* genes are similar to that of *AtMYB13 and AtMYB15*. As reported, *AtMYB13* and *AtMYB15* are involved in ABA-mediated responses to environmental signals [8], suggesting that the three *CpaMYBs* may be related to ABA-mediated stomatal closure in response to abiotic stresses. In addition, the expression of *Cpa001753* gene was clearly enhanced in both Salt and Salt+MeJA treatments (Figure 5) and a high occurrence of ABRE (ABA-responsive element) cis-acting element was detected in the promoters of *Cpa001753* (Figure 4), suggesting that *Cpa001753* may also participate in salt stress in the ABA-dependent pathway. However, further studies are required to reveal how the four *CpaMYB* genes work in ABA signaling pathways, and whether the regulation mechanism of the four *CpaMYB* genes is similar to that of *AtMYB13* or *AtMYB44* under abiotic stresses.

## 4. Materials and Methods

### 4.1. Plant Materials and Experiment Treatments

#### 4.1.1. Pot Experiment

In mid-October 2018, the seeds of *C. paliurus* were collected from a dominant tree (half-sib family) in Yanling, Hunan Province, China (26°31′ N, 113°49′ E). To overcome seed dormancy and improve the germination rate, exogenous GA3 (gibberellin A3) and stratification were used to treat the seeds [24]. In early April 2019, the germinated seeds were seeded in nonwoven containers (8.5 cm in diameter, 10.0 cm in depth) containing a mixture of perlites, rotten poultry excrement, soil, and peat = 2:2:2:4 (*v*/*v*/*v*/*v*). The contents of organic matter, total nitrogen, total phosphorus, total potassium, and pH were 73.3 g/kg, 72.35 g/kg, 2.19 g/kg, 9.55 g/kg, and 6.44, respectively. After two-month growth, the seedlings were transplanted to the greenhouse in Baima Experimental Base of Nanjing Forestry University (31°35′ N, 119°09′ E) for the two-week pretreatment. Salt treatments were conducted in mid-July 2019, and a completely random block design was used with 3 replicates per treatment and 6 plants per replicate.

Eight treatments were set up, including CK (control, distilled water), Salt (0.4%, M/V), MeJA (0.2 mM MeJA and distilled water), Salt + MeJA (0.4% NaCl and 0.2 mM MeJA), SNP (0.25 mM SNP and distilled water), Salt + SNP (0.4% NaCl and 0.25 mM SNP), NaHS (0.5 mM NaHS and distilled water), Salt + NaHS (0.4% NaCl and 0.5 mM NaHS). In order to avoid osmotic shock, NaCl solution was gradually added into the planting substrate 5 times within a week, until the concentration of NaCl solution reached 0.4%. However, the MeJA, SNP, and NaHS solutions were sprayed on the seedling leaves until completely wetting the leaves. The sample leaves were collected 30 days after the treatments. Three seedlings were selected from each treatment for sampling the leaves, while six intact and mature leaves were collected from the upper, middle, and lower parts of each sample seedling. After quickly frozen in liquid nitrogen, the leaves were stored at −80 °C for extracting total RNA.

Total RNA was extracted from *C. paliurus* leaves using Trizol reagent kit (Invitrogen, Carlsbad, CA, USA) and the quality was assessed using an Agilent 2100 Bioanalyzer (Agilent Technologies, Palo Alto, CA, USA). The cDNA fragments were purified with QiaQuick PCR extraction kit (Qiagen, Venlo, The Netherlands). The cDNA library was constructed for each sample using Illumina HiSeqTM 4000 by Gene Denovo Biotechnology Co. (Guangzhou, China). The raw sequencing data were submitted to the NCBI BioProject database under project number PRJNA799813.

#### 4.1.2. Hydroponic Experiment

In early October 2018, the *C. paliurus* seeds were collected from a dominant tree (half-sib family, Jinzhongshan No.11) in Jinzhongshan county, Guangxi Province, China (24°58′ N, 110°09′ E). After breaking seed dormancy, the germinated seeds were sown in nonwoven containers in April 2019. Uniform size seedlings were transplanted to polypropylene containers (50L) with 1/2-strength Hoaglands nutrient solution (pH 6.0 ± 0.2), then salt stress treatments were performed after two weeks after hydroponic transplanting. Four salt concentration treatments were designed—control (CK, 0 mM NaCl), 0.15% (LS, 25.7 mM NaCl), 0.30% (MS, 51.3 mM NaCl), and 0.45% (HS, 77.0 mM NaCl)—and arranged in a complete randomized block with three biological replicates. Each replication consisted of 8 seedlings. Similar to the pot experiment, the leaf samples were collected from three selected-sample seedlings 30 days after the treatments. After being quickly frozen in liquid nitrogen, the leaves were stored at −80 °C for total RNA extraction and transcriptome sequencing. The detailed information about hydroponic experiment was described in our previous study [3].

### 4.2. Gene Identification and Coding Protein Prediction

To obtain the protein database, transcriptome sequencing of *C. paliurus* was performed using the leaf samples collected from the pot experiment, and then the data were converted into group data and assembled with Trinity software to eliminate redundancy. The *A. thaliana MYB* sequences data were download from PlantTFDB database (http://planttfdb.cbi.pku.edu.cn/index.php accessed on 12 March 2021). Hidden Markov model (HMM) was established to search and obtain the HMM profile containing MYB domain (PF00249) [58], whereas MYB protein were identified from the database by software HMMER. All initial screening sequences were aligned and checked with the SMART software (http://smart.embl-heidelberg.de accessed on 19 March 2021), the Pfam database (http://pfam.xfam.org/ accessed on 22 March 2021), and the NCBI Batch CD-search (https://www.ncbi.nlm.nih.gov/Structure/bwrpsb/bwrpsb.cgi accessed on 25 March 2021). To keep all containing full length of the structure of MYB domain sequences, redundancy, repetition, and invalid sequences were removed. Then, the correct MYB sequences of *C. paliurus* were obtained. The ExPASy software (https://web.expasy.org/protparam/ accessed on 10 April 2021) was used to obtain basic physical and chemical characteristics of the *MYB* genes identified from the transcriptome sequencing data of *C. paliurus*, while the subcellular localization of these MYB proteins was predicted using the WoLF PSORT (https://wolfpsort.hgc.jp/ accessed on 20 April 2021) online tool.

### 4.3. Multiple Sequence Alignment and Phylogenetic Analysis

To group the R2R3-MYB proteins of *C. paliurus*, the phylogenetic relationships between *C. paliurus* and *A. thaliana* were analyzed, and the established phylogenetic tree consisted of 120 R2R3-MYB proteins from *A. thaliana* and 69 R2R3-MYB proteins from *C. paliurus*. Multiple sequence alignment (MSA) of *C. paliurus*. and *A. thaliana* MYB-domain-containing proteins was performed using Clustal X 2.11 software [59], while MEGA X (version 6.0) software [60] was used to conduct phylogenetic analysis using the maximum likelihood method with 1000 bootstrap replicates.

### 4.4. Exon–Intron Structural and Conserved Motif Analysis

Based on the transcriptome sequencing data, the CDS sequences of 69 *R2R3-MYB* genes of *C. paliurus* were obtained. The Gene Structure Display Server (GSDS) online tool (http://gsds.cbi.pku.edu.cn/ accessed on 27 April 2021) was adopted to obtain the exon–intron structures of *Cpa MYB* genes, whereas fifteen conserved motifs were obtained using the online software MEME (http://MEME-suite.org/tools/MEME accessed on 8 May 2021) by setting the optimal sequence width to 30–50 and the parameters Zero to one occurrences per sequence. Furthermore, to visualize the conserved motifs with TBtools (version 1.098696) software [61], the associated mast file was then downloaded from MEME.

### 4.5. Promoter Cis-Acting Element Prediction

BEDtools (version 2.25.0) [62] was used to extract a 2000-bp sequence upstream of the transcription start site of *CpaR2R3-MYB* genes from transcriptome sequencing data. Afterwards, the PlantCARE (https://bioinformatics.psb.ugent.be/webtools/plantcare/html/ accessed on 23 May 2021) online tool was adopted to identify the motifs present in the promoter sequences.

### 4.6. Expression Profile under Salt Stress and qRT-PCR Analysis

In the pot experiment, transcript abundance was determined based on fragments per kilobase of transcript per million mapped reads (FPKM) values. The expression profile data of *C.* *paliurus* were obtained from the transcriptome sequencing in the treatments of CK, Salt, Salt + SNP, Salt + MeJA, and Salt + NaHS. The TBtools software was used to generate the heat map. Among 17 *MYB* genes identified from phylogenetic trees that may be related to abiotic stress [23,24,25,63,64], 10 genes were selected for validation by qRT-PCR.

Trizol reagent kit (Invitrogen, Carlsbad, CA, USA) was used to extract RNA from 15 samples of the 5 treatments (CK, Salt, Salt + SNP, Salt + MeJA, and Salt + NaHS); subsequently, MonScript RTIII All-in-One Mix with dsDNase kits (Monad, Nanjing, China) was used to acquire cDNA. Primer Premier 6.0 (Premier Biosoft International, Palo Alto, CA, USA) was used to design qRT-PCR primers for 10 genes (Table S3). SYBR Premix Ex Taq kit (Takara Biotechnology, Dalian, China) was applied to conduct qRT-PCR analysis. The cDNA diluted 20 times and an 18sRNA gene were chosen as the template and the internal standard, respectively [65]. PCR reaction conditions were as 95 ℃ for 3 min; denaturation 5 s at 95 °C; 60 °C for 30 s; 40 cycles. Three technical and three biological replicates were used for each sample. After reaction, the relative expression levels of target gene and internal reference gene were calculated with the 2^−ΔΔCT^ method [66].

In the hydroponic experiment, transcriptome sequencing analysis was conducted using leaf samples collected from *C. paliurus* seedlings after 30 days of salt treatments. The RPKM data of *MYB* genes in four salt concentrations (namely, CK, LS, MS, and HS) were obtained from the transcriptome data. The TBTOOLS software was used to generate the heat map.

### 4.7. Statistical Analysis

One-way analysis of variance (ANOVA) was conducted to identify significant differences in the related gene expression among the salt treatments, followed by Tukey’s test for multiple comparisons. All statistical analyses were performed using IBM SPSS Statistics Version 22 software package (SPSS Inc., IBM Company Headquarters, Chicago, IL, USA). Data were presented as means ± standard deviation (SD).

## 5. Conclusions

In this study, 211 *CpaMY*B genes and their corresponding protein sequences were identified from the full-length transcriptome, while 153 MYB protein sequences including 69 R2R3-MYB sequences of *C. paliurus* with typical complete MYB domain were obtained. Combining the results from both the pot and hydroponic experiments, we infer that four *R2R3-MYB* genes (*Cpa001753*, *Cpa115159*, *Cpa107784*, and *Cpa112016*) may participate in the salt stress regulation of *C. paliurus* via phytohormone signals. This study provides a basis for further functional characterization of R2R3-MYB TFs in *C. paliurus*; however, further studies are required in order to better elucidate the regulation mechanism of these four *R2R3-MYB* TFs to salt stress in this species.

## Supplementary Materials

The following supporting information can be downloaded at: https://www.mdpi.com/article/10.3390/ijms23073429/s1.
