# Supplementary material for: Identification and Expression Analysis of R2R3-MYB Family Genes Associated with Salt Tolerance in Cyclocarya paliurus
Source: Int J Mol Sci. 2022 Mar 22;23(7):3429. doi: 10.3390/ijms23073429 (PMC8998414; doi:10.3390/ijms23073429)
Supplement: Supplementary file 1 [file ijms-23-03429-s001.zip › ijms-1635453-supplementary.pdf]

Supplementary Materials

Table S1. Physical and chemical property prediction and location of R2R3-MYB family in *C. paliurus*

Gene ID	Amino acids (aa)	Molecular weight (Mw:Da)	Isoelectric point (pI)	Subcellular localisation
Cpa001080	303	34196.55	7.01	nucleus
Cpa001753	367	41688.57	5.43	nucleus
Cpa002553	265	30101.94	10.53	nucleus
Cpa010180	351	40451.84	8.32	nucleus
Cpa015189	304	32845.89	8.69	nucleus
Cpa030403	317	35692.78	5.47	nucleus
Cpa030404	342	38425.84	5.57	nucleus
Cpa033006	108	12606.55	9.20	nucleus
Cpa033987	319	34508.02	6.96	nucleus
Cpa035247	266	30919.94	6.38	nucleus
Cpa035470	196	22367.81	9.75	nucleus
Cpa035471	178	20344.31	9.72	nucleus
Cpa035472	216	24275.16	6.25	nucleus
Cpa036268	175	20937.19	9.37	nucleus
Cpa036601	269	30255.79	5.68	nucleus
Cpa036603	264	30008.62	6.17	nucleus
Cpa038889	338	37747.42	5.90	nucleus
Cpa038890	346	38599.42	8.37	nucleus
Cpa038891	356	39517.34	8.56	nucleus
Cpa040502	371	41747.07	5.86	nucleus
Cpa041489	261	29907.10	5.89	nucleus
Cpa042286	316	35180.76	5.79	nucleus
Cpa043908	441	49362.77	7.14	nucleus
Cpa044885	249	28852.68	8.50	nucleus
Cpa050749	362	39234.79	5.36	nucleus
Cpa050782	265	39143.83	4.86	nucleus
Cpa050783	306	35086.14	5.24	nucleus
Cpa051074	379	42012.03	8.15	nucleus
Cpa051147	227	26217.33	6.17	nucleus
Cpa056821	324	35750.98	6.83	nucleus
Cpa056977	497	54628.02	5.49	nucleus
Cpa058837	235	25920.69	8.57	nucleus
Cpa059447	416	46637.08	5.16	nucleus
Cpa062832	334	37231.40	7.58	nucleus
Cpa063804	210	23777.00	8.92	nucleus
Cpa063805	244	27766.31	6.70	nucleus
Cpa064937	399	44735.09	5.87	nucleus
Cpa065180	313	36124.32	5.33	nucleus

Cpa067029	356	40852.72	9.35	nucleus
Cpa067221	417	47819.03	10.08	nucleus
Cpa067744	376	42212.37	7.22	nucleus
Cpa067763	345	39017.96	5.09	nucleus
Cpa068515	378	42976.68	8.84	nucleus
Cpa068516	374	42654.80	9.41	nucleus
Cpa074775	271	30622.45	5.07	nucleus
Cpa083036	456	50626.74	5.80	nucleus
Cpa087248	373	41965.96	5.59	nucleus
Cpa093839	556	60299.14	5.02	nucleus
Cpa094184	282	31589.43	9.56	nucleus
Cpa095422	206	23713.23	9.30	nucleus
Cpa095998	358	40788.27	5.41	nucleus
Cpa098838	248	27671.37	8.87	nucleus
Cpa100953	251	29573.16	8.18	nucleus
Cpa100954	252	29494.05	8.79	nucleus
Cpa104103	370	41813.68	5.58	nucleus
Cpa106610	324	36698.07	5.72	nucleus
Cpa107784	281	31708.26	5.07	nucleus
Cpa108647	478	54115.60	6.02	nucleus
Cpa108726	231	25971.50	9.14	nucleus
Cpa111949	288	32265.24	6.15	nucleus
Cpa112016	281	31684.23	4.90	nucleus
Cpa113885	318	34679.10	7.01	nucleus
Cpa115159	225	25677.32	9.61	nucleus
Cpa117362	314	35305.91	9.24	nucleus
Cpa120104	389	42573.16	5.63	nucleus
Cpa120105	399	43977.80	6.11	nucleus
Cpa122160	321	35797.25	5.92	nucleus
Cpa124579	443	47883.85	5.28	nucleus
Cpa124871	361	40542.45	6.13	nucleus

Table S2. classification of the phylogenetic tree and function prediction of each group

Group	Subgroup	Function prediction
C1	S3	Involved in biotic stress responses
C2	S2	Involved in trichome development
C3	S2	Involved in development of epidermal cells
C4	S2	Involved in cell morphogenesis, cuticle pattern formation，regulation of cutin biosynthetic process and trichome branching
C5	S1	Involved in lignin biosynthesis
C6	S1	Involved in scw synthesis
C7	S3	Involved in cold stress tolerance
C8	S1	Involved in scw synthesis
C9	S3	Involved in environmental stress responses
C10	S2	Involved in cell morphogenesis, cuticle pattern formation，regulation of cutin biosynthetic process and trichome branching
C11	S1	Involved in anthocyanin biosynthesis
C12	S2	Involved in formation of the seed coat and underlying endospermlayers
C13	S1	Involved in flavonol biosynthesis
C14	S1	Involved in lignin deposition and anthocyanin biosynthesis
C15	S2	Involved in development of epidermal cells
C16	S2	Involved in far-red light responses
C17	S1	Involved in scw synthesis
C18	S1	Involved in lignin deposition
C19	S1	Involved in biosynthesis of proanthocyanidins
C20	S2	Involved in root,anther and pollen development
C21	S3	Involved in stress responses
C22	S2	Involved in anther and stamen development
C23	S2	Involved in root development
C24	S2	Involved in specification of the leaf proximodistal axis
C25	S2	Involved in stomata development
C26	S3	Involved in stress responses
C27	S1	Involved in lignin xylan and cellulose biosynthesis
C28	S2	Involved in embryogenesis (seed, endosperm and cell differentiation)
C29	S2	Involved in regulatory mechanism of glucose metabolism

Table S3 Information of primer sequences

Gene ID	Primer sequences	Length/bp
18s-FP	AGTATGGTCGCAAGGCTGAAA	21
18s-RR	CAGACAAATCGCTCCACCAA	20
Cpa115159-FP	CCAACCAAACGCAACACAATTAAAA	25
Cpa115159-RP	GACGAACGGACAATGAGATAAAGA	24
Cpa112016-FP	TAACTAATCTACTTTTTCCCTATAA	25
Cpa112016-RP	TGTTTGGCGGTTTGGAGG	18
Cpa095998-FP	TTGTCATGCCCTTATGGTTTATTCG	25
Cpa095998-RP	GGATTGTTTGCGTACTCGTGAT	22
Cpa107784-FP	TCTGTGTCGATCTTTTTTTTACGTA	25
Cpa107784-RP	CCAAGCCCAAAAGCCAGA	18
Cpa058837-FP	AACCACTGGAACTCCACTCTGAAGA	25
Cpa058837-RP	GGTCGTCGTCCTCCTCCATT	20
Cpa094184-FP	GGAGGACCAGATCATTGTTAAGGCC	25
Cpa094184-RP	GATTTCTTGTGCGGTCGTGAT	21
Cpa015189-FP	TTACTGAGTCTCTCTCTTCCCGGAT	25
Cpa015189-RP	GTAGGACTCCCAGCCCGATT	20
Cpa033987-FP	GATCCGATCCATTTCGTTTTTACAA	25
Cpa033987-RP	GTCCAACCAACCCATTTTCG	20
Cpa113885-FP	CGGGCGTGGGCACGAACAATGGTAT	25
Cpa113885-RP	TACGCCAGAGGAGGACGATAC	21
Cpa001753-FP	CCGGCCACCATGAAAACCCAAACTT	25
Cpa001753-RP	CGAGGTGCTGAAGTGGGTAAA	21
